# Liver abscess caused by the ingested foreign body without sign of gastrointestinal perforation: A case report

**DOI:** 10.1016/j.radcr.2023.09.028

**Published:** 2023-10-06

**Authors:** Le Huu Nhat Minh, Luu Thi Kim Han, Nguyen Viet Hau, Nguyen Anh Kiet, Tang Tuan Phong, Nguyen Khanh Duong, Phan Thi Hoang Yen, Nguyen Xuan Vinh, Hien Quang Nguyen, Nguyen Quoc Khanh Le

**Affiliations:** a International Ph.D. Program in Medicine, College of Medicine, Taipei Medical University, Taipei 110, Taiwan; b AIBioMed Research Group, Taipei Medical University, Taipei 110, Taiwan; c Research Center for Artificial Intelligence in Medicine, Taipei Medical University, Taipei 110, Taiwan; d Emergency Department, University Medical Center, Ho Chi Minh City, Vietnam; e Faculty of Medicine, University of Medicine and Pharmacy, Ho Chi Minh City, Vietnam; f Cardiovascular Research Department, Methodist Hospital, Merrillville, IN 46410, USA; g Professional Master Program in Artificial Intelligence in Medicine, College of Medicine, Taipei Medical University, Taipei 110, Taiwan; h Translational Imaging Research Center, Taipei Medical University Hospital, Taipei 110, Taiwan

**Keywords:** Liver, Abscess, Ingested, Foreign body, CT, Radiology, Surgery

## Abstract

The ingested foreign body is a very unusual etiology of liver abscess. This clinical scenario is infrequently reported in the literature. A 66-year-old male patient presented to the hospital because of abdominal pain along with 7 days of right upper quadrant pain and intermittent low-grade fever. He was living in an epidemiological area of Fasciola infection. Physical examination showed right hypochondria tenderness without guarding or rebounding. Laboratory results were significant for leukocytosis, predominant neutrophils, and increased inflammatory markers. The liver function tests were within normal limits. Abdominal ultrasonography and CT scan were consistent with a hepatic abscess spread from segment 4B to segment 3. The patient was preliminarily diagnosed with a parasitic hepatic abscess. After management with fluid infusion and antibiotics, the patient was discharged in stable condition. Two weeks later, on the follow-up visit, the patient reported intermittent low-grade fever had persisted. After consulting the CT scan, an abnormal high-attenuation linear structure was identified inside the liver lesion, which is suspected of being a foreign body. Laparoscopic surgery was performed, and a fishbone was removed from the abscess cavity. Perforation was not found in the stomach, duodenum, or in the bowel. One week later, their condition was fully resolved. Liver abscess due to a foreign body should be suspected when a patient has radiology findings suggestive of an abscess, but the clinical presentation does not indicate the common etiologies. Meticulous observation on abdominal CT scans or ultrasonography can help with diagnosis and guide treatment.

## Background

Liver abscess is not a rare disease but can be caused by many different etiologies [1,2]. However, explaining the origin which induces liver abscess is not always straightforward, because clinical symptoms are often nonspecific [2]. Especially in the endemic area of fascioliasis, the parasitic hepatic abscess may be a suggestive diagnosis but also can become a confounding factor [1]. We report a case of liver abscess caused by a gastrointestinal foreign body without symptoms of intestinal perforation. This is a very unusual etiology of liver abscess and is infrequently reported in the literature [3]. Definitive diagnosis and surgical intervention play a key role in prognosis.

## Case presentation

A 66-year-old male patient presented to the emergency department because of abdominal pain. On admission, the patient was oriented, the blood pressure was 90/60 mmHg, the heart rate was 89 beats per minute, the temperature was 36.7°C, the respiratory rate was 18 breaths per minute, and oxygen saturation was 98% on room air. During the past 7 days, they had dull pain in the right hypochondriac region and intermittent low-grade fever. The patient had not vomited or experienced any episode of diarrhea.

### Patient's history

The patient resided in an area with a high incidence of Fasciola infection. The patient had a non-smoking history, consumed alcohol occasionally, and did not engage in recreational drug use. Their past medical history was unremarkable, except for a hernia repair performed 10 years prior in the inguinal region. The patient was immunocompetent and tested negative for hepatitis B, hepatitis C, and human immunodeficiency virus (HIV).

### Physical exam and pertinent laboratory values

At admission, the physical examination revealed mild tenderness in the right hypochondriac region without guarding or rebound tenderness. No signs of jaundice were observed. On their first admission, laboratory results were significant for leukocytosis (12.3 G/L) with 75.3% of neutrophils, 13.8% of lymphocytes, 1.09% of eosinophils, elevated C-reactive protein level (79.8 mg/dL), and increased fibrinogen level (6.88 g/L). The hemoglobin level was 129 g/L. The liver enzyme and serum bilirubin were within normal limits. Immunodiagnosis of amebiasis and fascioliasis was not reactive.

### Imaging findings

**Fig. 1:**
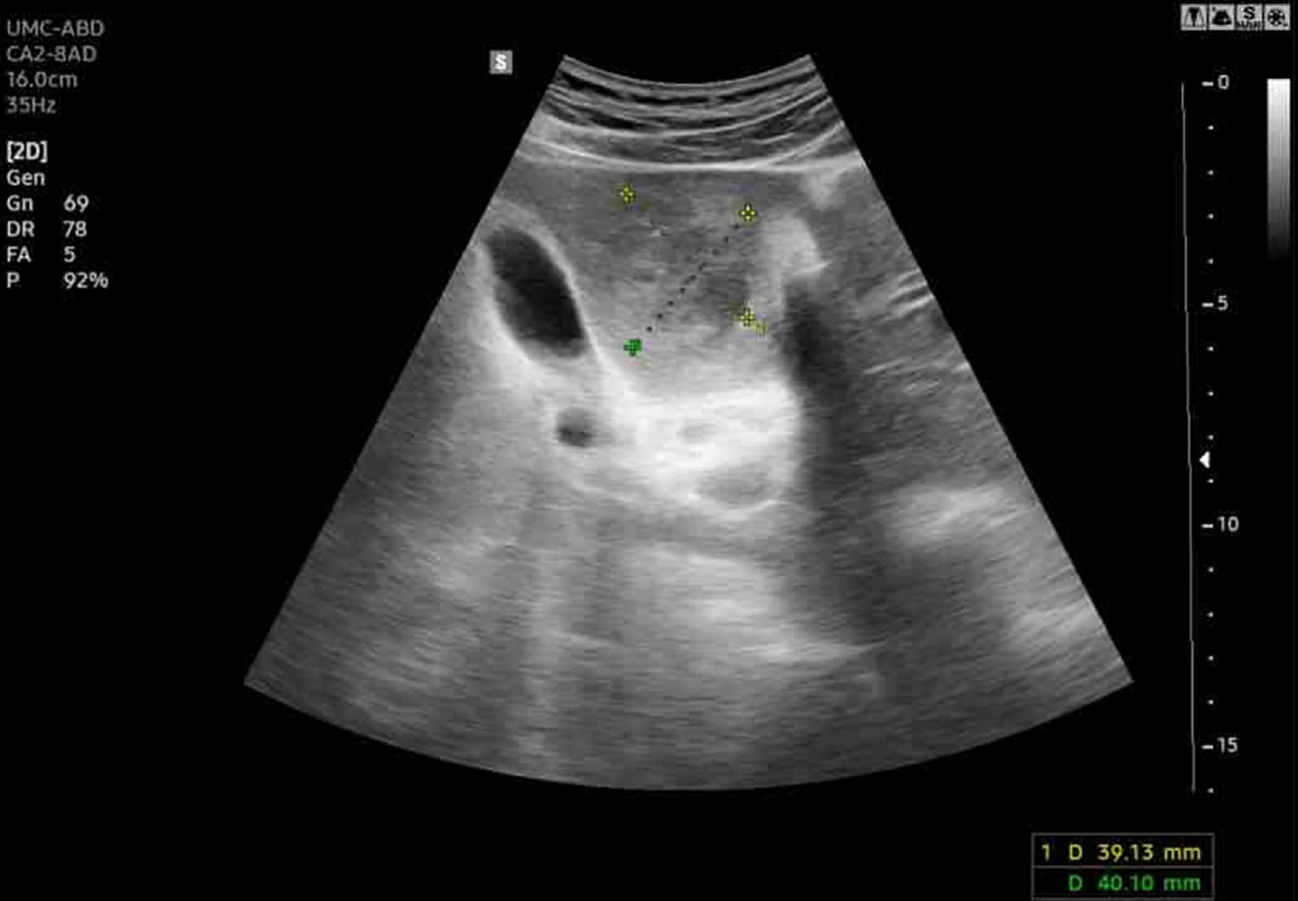
Ultrasonography shows a poorly demarcated, heterogeneous lesion in segment 4B of the liver.

**Fig. 2:**
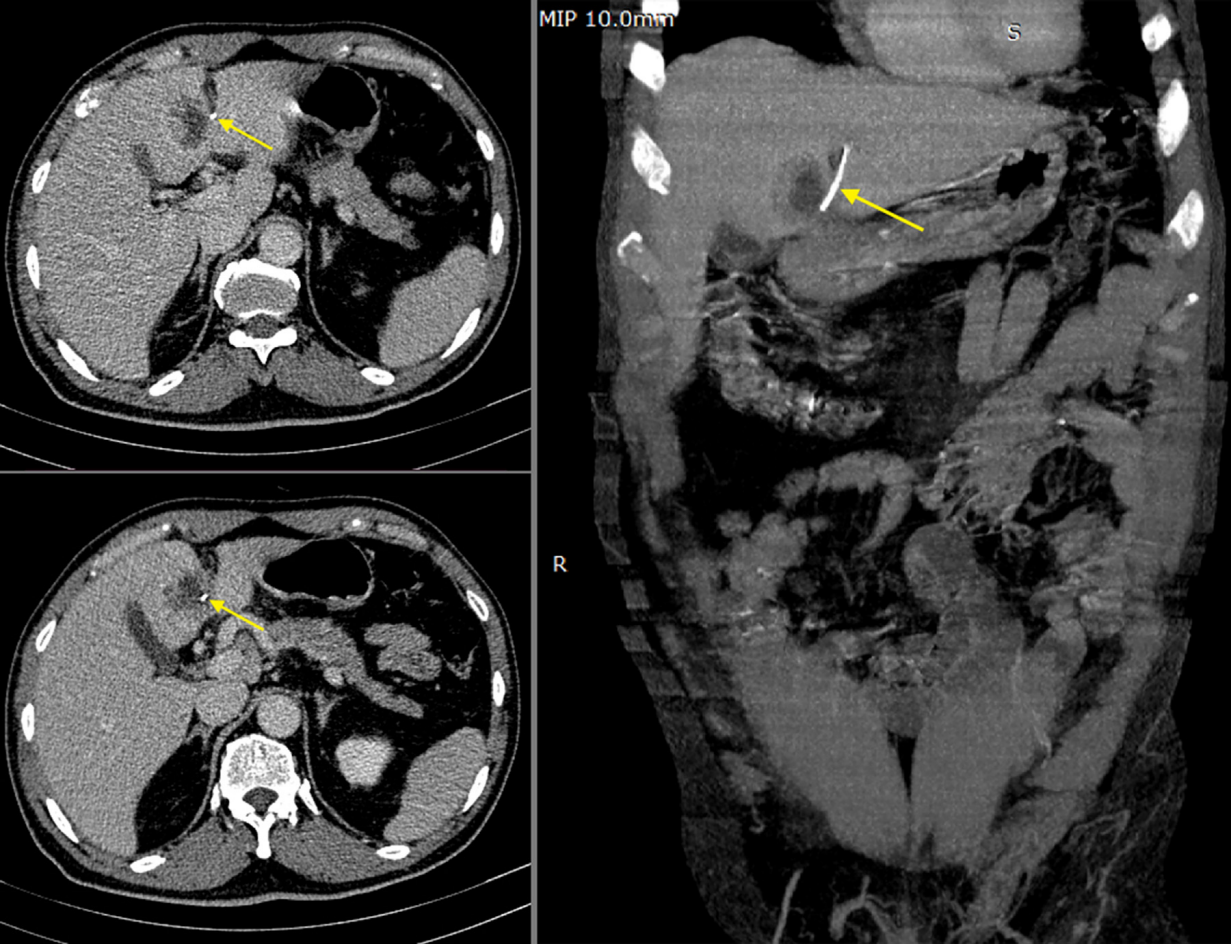
CT scan of the abdomen showed an abscess with a linear foreign body (arrow) in segment 4B and segment 3 of the liver.

Ultrasonography revealed a poorly demarcated, heterogeneous lesion in segment BE (Fig. 1). Centrally, the lesion had hypoechoic areas which demonstrated a liquefied necrotic process. No presence of abdominal fluid was recorded. Abdominal CT scan with intravenous contrast showed a localized lesion that spread from segment BE to segment 3 with the largest diameter of 38 mm (Fig. 2). The lesion appeared peripherally enhancing, with irregular margins, centrally hypoattenuating with incomplete septal formation, and circumferential perfusion abnormalities were visible. At that time, the parasitic hepatic abscess was a preliminary diagnosis based on the radiology findings and epidemiological factors. The patient was managed with fluid infusion, anthelmintics, and antibiotics, including triclabendazole, cefixime, and metronidazole, before being discharged in stable condition on the same day.

On the follow-up visit 2 weeks later, the patient reported declining abdominal pain, but the intermittent fever persisted with shaking chills. On physical examination, the patient continued having mild right hypochondria tenderness. White blood cell count increased to 29.62 G/L with predominant neutrophils (94.8%). The procalcitonin level was 53.9 ng/mL (normal value < 0.5 ng/mL). The Hemoglobin level decreased to 107 g/L. Serum gamma-glutamyl transferase was elevated (245 U/L). After consulting the CT scan, an abnormal high attenuation linear structure located inside the lesion in the segment by of the liver was identified, which is suspected of a foreign body. There was no intra or extraperitoneal gas or fluid. The patient was diagnosed with a liver abscess caused by a foreign body.

### Surgical results

**Fig. 3:**
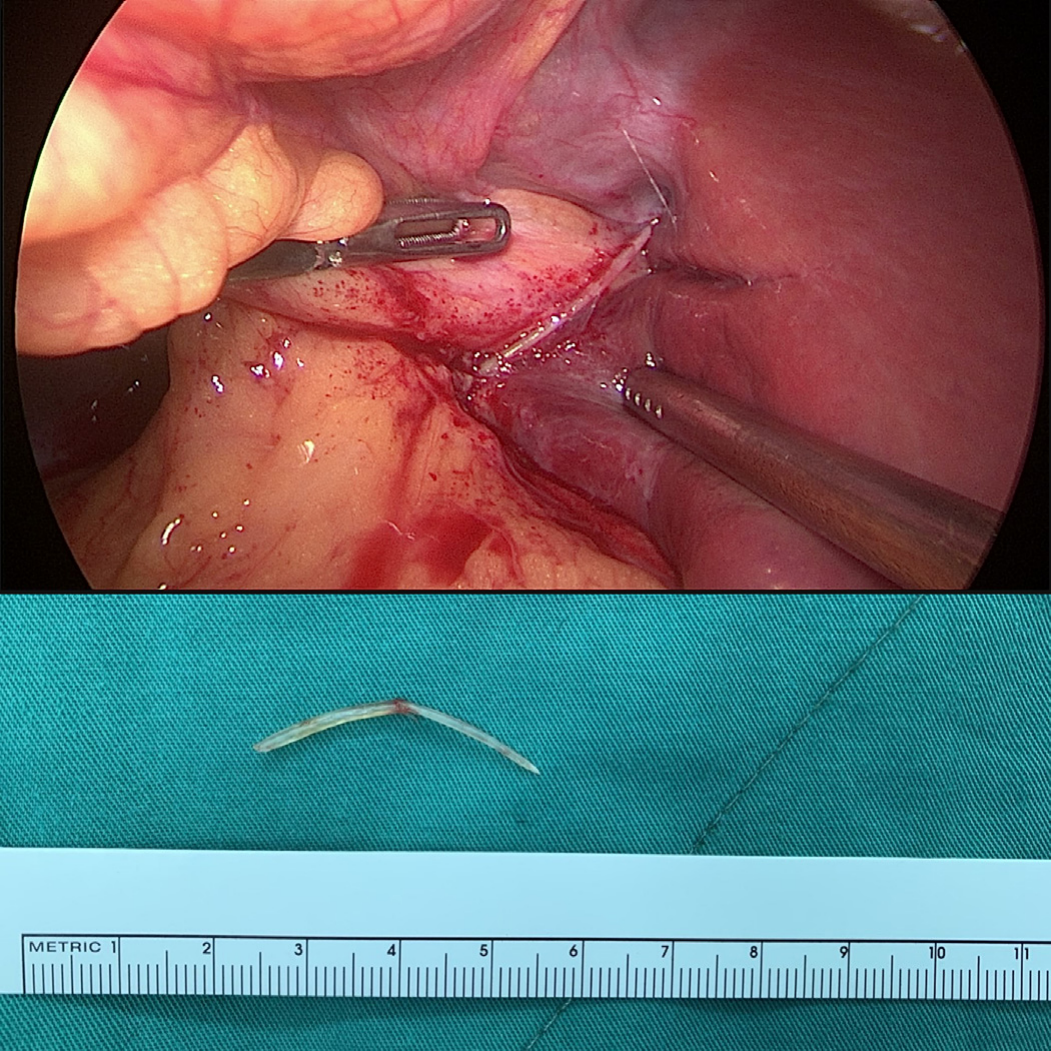
Laparoscopic surgery removed a fishbone 3 cm in size from the liver, no perforation was noted.

A laparoscopic surgical intervention was undertaken to extract the ingested foreign body and drain the liver abscess. Intraoperatively, a 3 cm fishbone was discovered embedded within the liver, while no evidence of perforation was identified in the stomach or duodenum (Fig. 3).

### Final diagnosis

Blood culture was positive for *Streptococcus gordonii*, and surgical abscess drainage culture was negative. Postoperatively, the patient had no fever, white blood cell count and other inflammatory markers gradually decreased. One week later, their condition was fully resolved. The final diagnosis is a liver abscess caused by the ingested foreign body without the sign of gastrointestinal perforation.

## Discussion

When foreign bodies are ingested, they can sometimes penetrate the gastrointestinal tract and cause perforation, leading to the development of an intraperitoneal abscess. However, it is very rare for a hepatic abscess to form because of ingested foreign bodies. Diagnosis of such cases can be challenging due to the presence of a wide variety of non-specific symptoms. Additionally, patients are often unaware of the ingestion and do not exhibit signs of gastrointestinal perforation. As a result, it can be difficult to determine the duration between the ingestion of the foreign body and the formation of the abscess. Furthermore, the migration of the foreign body may remain silent until it reaches the liver and causes an abscess to form. In some instances, the perforation may lead to the formation of an intraperitoneal abscess before the foreign body even penetrates the liver capsule [4], [5]–6]. In our case, the perforation was completely occluded, resulting in no physical or radiologic findings, and was not discovered during laparoscopic surgery.

The prevailing clinical manifestations of the condition under consideration are pyrexia and non-specific, persistent abdominal discomfort. These symptoms reflect the body's systemic response to infection or abscess formation. Patients may present with sepsis or septicemia that is refractory to antibiotics, as documented in this case [7], [8]–9]. In addition, routine laboratory investigations may reveal elevated levels of inflammatory markers, without any notable abnormalities in liver function tests.

While hepatic abscesses are often readily detected through radiologic imaging, the presence of a foreign body can be easily overlooked if clinical physicians and radiologists are not suspicious of this etiology. Following the American College of Radiology (CAR) Appropriateness Criteria and EURO-2000 Guidelines, ultrasonography has been established as a valuable initial imaging modality, demonstrating high sensitivity in the detection of liver abscesses [10]. Additionally, abdominal ultrasonography is widely regarded as the preferred diagnostic method for both amebic liver abscess and pyogenic liver abscess, with reported sensitivities of 90% or greater for amebic liver abscess, and 85.8% for pyogenic liver abscess, respectively [11,12]. However, the echoic characteristics of these lesions are nonspecific, and detecting small foreign bodies can be challenging during the initial assessment. In our case, the ingested foreign body was anatomically located in segment be, predominantly in a caudal-cranial direction. Therefore, despite standard liver ultrasound planes, the object was predominantly cut in cross-section and was barely perceptible, appearing as a small dot. The correct longitudinal section of the object was only identified when there was a reference CT scan to adjust the ultrasound plane. X-rays may be helpful, but only for radiopaque objects. In our case, the foreign body was a fishbone, and radiographs were unable to provide a clear diagnosis. Additionally, the absence of indirect signs of gastrointestinal perforation, such as free abdominal gas or fluid, further complicated the diagnosis.

Abdominal CT scan plays a crucial role in definitive diagnosis and treatment planning. Contrast-enhanced abdominal CT is particularly sensitive in detecting inflammatory changes in the peritoneum and foreign bodies. However, in our patient, a delay of one week from the onset of symptoms until presentation led to the formation of an abscess and closure of the perforation. As a result, the signs of peritoneal inflammation changes, such as fatty stranding and enhancement, were absent. Furthermore, the abscess appearance was similar to that of a Fasciolosis-induced liver abscess, given that the patient resided in an endemic area. Hence, the likelihood of rare etiologies was low [13]. Meticulous observation, combined with multiplanar reconstruction, was instrumental in accurately identifying the size and location of the foreign body, leading to a definitive diagnosis and guidance for subsequent surgery.

The blood culture results of the patient are consistent with the literature, revealing an infection caused by *S. gordonii*, a Gram-positive commensal bacterium typically encountered in the oral cavity, skin, and intestine. This finding strongly suggested that the foreign body served as a vehicle for bacterial transport into the liver, rather than acting as a nidus for transient bacteria in the blood to attach and proliferate. In some cases, Gram-negative, anaerobic, or multiple organisms have been isolated [4,14]. Therefore, the administration of broad-spectrum antibiotics was necessary.

Surgical intervention to remove the foreign body is a critical component of treatment. Endoscopic or percutaneous removal can be considered alternative option [9]. In addition to foreign body removal, abscess drainage and antibiotics are also essential components of treatment. In cases involving granulomatous formation, a partial liver resection may be recommended [6]. If not promptly diagnosed and treated, complications such as septic shock can be life-threatening [15].

## Conclusion

Liver abscess resulting from foreign body ingestion is an uncommon but serious condition that requires attention when radiological findings suggest abscess, but common causes are not evident in clinical presentation. Abdominal CT imaging is a vital diagnostic tool for these cases as it provides detailed visualization of the foreign body and any associated complications. Patients with this condition may manifest septicemia or sepsis that does not improve with antibiotic therapy, rendering surgical intervention necessary. Therefore, surgical removal of the foreign body is the preferred therapeutic approach in this situation.

## Patient consent

Written informed consent for the publication of this case report was obtained from the patient.

## Data Availability

All data generated or analyzed during this study are included in this article.
